# Early Versus Late Tracheostomy in Spontaneous Intracerebral Hemorrhage

**DOI:** 10.7759/cureus.24059

**Published:** 2022-04-12

**Authors:** David R Hallan, Christopher Simion, Elias Rizk

**Affiliations:** 1 Neurosurgery, Penn State Health Milton S. Hershey Medical Center, Hershey, USA

**Keywords:** length of stay, nosocomial infection, mortality rate, outcomes, hemorrhagic stroke, spontaneous intracerebral hemorrhage, tracheostomy, neurosurgery

## Abstract

Introduction: Recent literature supports early tracheostomy (<=7 days) over delayed tracheostomy (>7 days-3 months) to improve overall clinical outcomes for patients admitted with an acute head injury. There is conflicting evidence for the same in hemorrhagic stroke. Using a multi-institutional database, we explored this question in nontraumatic spontaneous intracerebral hemorrhage (sICH) patients.

Methods: We used a de-identified database network (TriNetXʼs Research Network) to gather information on early tracheostomy (<=7 days) and late tracheostomy (>7d-3 months) in sICH patients. After accounting for the most common comorbidities, we explored the impact of this intervention on multiple patient outcomes including intensive care unit (ICU) length of stay, pneumonia, and mortality at 30, 90, and 365 days.

Results: After propensity score matching, a total of 1210 patients were identified for both early tracheostomy (cohort 1) and late tracheostomy (cohort 2) cohorts. The 30-day survival rate was 0.9287 in cohort 1 vs 0.9536 in cohort 2, with a risk difference of 2.39% (95% confidence interval (CI) 0.557%-4.23%; relative risk (RR) 1.54, 95% CI (1.10-2.15); OR 1.577, 95% CI (1.11-2.24); p = 0.006). The 90-day and 365-day end-point survival rates were not statistically different between cohorts. ICU level of care codes were billed an average of 9.76 (SD 8.964) times in cohort 1 vs 14.618 (SD 11.851) in cohort 2 (p<0.0001). At 365 days, there were no differences between the two groups for pulmonary embolism, myocardial infarction, deep venous thrombosis, palliative care consultation, and percutaneous endoscopic gastrostomy tube placement. Cohort 1 had decreased incidence of pneumonia with 665 (54.95%) patients compared to cohort 2 with 725 (59.91%) (RR 0.917, 95% CI (0.856-0.983), OR 0.816, 95% CI (0.695-0.95), p = 0.013).

Conclusion: Early tracheostomy in sICH patients was associated with decreased pneumonia risk, decreased length of ICU care, and no difference in mortality at 90 and 365 days.

## Introduction

Spontaneous intracerebral hemorrhage (sICH) is defined as nontraumatic bleeding into the brain parenchyma which can extend to the ventricles and subarachnoid space [1]. It is the second most common subtype of stroke [2], responsible for case-fatality ranges of 35% at 7 days to 59% at 1 year [3,4]. Those who recover are often left with a disability; less than 40% of patients regain functional independence [2]. In-hospital complications are known to be correlated with increased length of stay. Most available stroke data concerning in-hospital complications pertain to ischemic stroke, with few focusing on sICH [5]. Prior literature by Rizk et al. found a complex relationship with tracheostomy timing and outcomes in neurotrauma patients that suggested a strategy of early tracheostomy (< 7 days) resulted in better overall clinical outcome, including functional outcome, versus late tracheostomy [6]. Given this, we sought to observe whether a similar strategy in nontraumatic sICH would benefit from early tracheostomy.

## Materials and methods

This was a retrospective comparative case-control study. We used a de-identified database network (TriNetX) to retrospectively query via the International Classification of Disease (ICD-10) and current procedural terminology codes to evaluate all patients with a diagnosis of spontaneous intracerebral hemorrhage who received a tracheostomy within 7 days (cohort 1) versus 8 days-3 months (cohort 2). Data came from 57 healthcare organizations (HCOs) spanning six countries (the United States, United Kingdom, Germany, Italy, Israel, and Singapore). Data includes demographics, diagnoses, medications, laboratory values, genomics, and procedures. The identity of the HCOs and patients is not disclosed to comply with ethical guidelines against data re-identification. Because of the databaseʼs federated nature, an Institutional Review Board (IRB) waiver has been granted. The data is updated daily. Previous literature informed our use of this database and its validity, and the networkʼs exact details have been previously described [7-10]. The diagnosis was based on ICD-10 codes and procedural codes via Common Procedural Terminology (CPT) codes. The index date was set at the date of sICH.

Medical information including age at index date, as well as sex, race, and comorbidities of hypertension, acute kidney injury, diabetes, ischemic heart disease, heart failure, atrial fibrillation, disorders of lipoprotein metabolism and other lipidemia, obesity, history of nicotine dependence, chronic respiratory disease, cirrhosis, alcohol abuse or dependence, and peripheral vascular disease, recorded up to the date of the index date. Our primary endpoint was mortality at 1-year. Secondary endpoints included percutaneous endoscopic gastrostomy (PEG) placement, engagement of palliative care, days spent in the intensive care unit (ICU), pneumonia/pneumonitis, pulmonary embolism (PE), myocardial infarction (MI), and deep venous thrombosis (DVT). Analysis was performed using unmatched and propensity score-matched cohorts, with the greedy-nearest neighbor algorithm with a caliper of 0.1 pooled standard deviations. Chi-square analysis was performed on categorical variables. Significance was defined as a p-value less than 0.05.

## Results

After propensity score matching, a total of 1,210 patients were identified for both early tracheostomy (cohort 1) and late tracheostomy (cohort 2) cohorts. Age at index was 50.31+-18.69 years and 50.13+-19.14 years for cohorts 1 and 2, respectively. 65.54% of cohort 1 were male, versus 67.36% in cohort 2. 58.099% vs 59.256% of patients were white, 22.314% vs. 21.322% were black or African American, and 17.190% vs. 17.273% were of unknown race. Baseline demographics and characteristics are shown in Table 1.

**Table 1:** Baseline demographics and characteristics after propensity score matching

		Before Matching			After Matching		
Code	Diagnosis	Cohort 1, n (%)	Cohort 2, n (%)	Std diff.	Cohort 1, n (%)	Cohort 2, n (%)	Std diff.
AI	Age at Index	50.33 (100)	53.45 (100)	-	50.31 (100)	50.13 (100)	-
M	Male	794 (65.57)	2162 (58.13)	0.15	793 (65.54)	815 (67.36)	0.039
2106-3	White	704 (58.13)	2124 (57.11)	0.021	703 (58.09)	717 (59.26)	0.023
F	Female	417 (34.43)	1557 (41.87)	0.15	417 (34.46)	395 (32.65)	0.039
2054-5	Black or African American	270 (22.29)	978 (26.29)	0.093	270 (22.31)	258 (21.32)	0.024
2131-1	Unknown Race	208 (17.18)	534 (14.36)	0.077	208 (17.19)	209 (17.27)	0.0022
2028-9	Asian	24 (1.98)	66 (1.78)	0.015	24 (1.98)	24 (1.98)	0
I10-I16	Hypertensive diseases	430 (35.51)	1986 (53.40)	0.37	430 (35.54)	422 (34.88)	0.014
R40	Somnolence, stupor and coma	326 (26.92)	1173 (31.54)	0.10	326 (26.94)	313 (25.87)	0.024
R13	Aphagia and dysphagia	270 (22.29)	1142 (30.71)	0.19	270 (22.31)	257 (21.24)	0.026
N17-N19	Acute kidney failure and chronic kidney disease	180 (14.86)	887 (23.85)	0.23	180 (14.88)	196 (16.19)	0.037
E08-E13	Diabetes mellitus	149 (12.30)	713 (19.17)	0.19	149 (12.31)	153 (12.65)	0.01
E78	Disorders of lipoprotein metabolism and other lipidemias	132 (10.90)	691 (18.58)	0.22	132 (10.91)	129 (10.66)	0.0079
F17	Nicotine dependence	119 (9.83)	468 (12.58)	0.088	119 (9.84)	122 (10.08)	0.0083
I20-I25	Ischemic heart diseases	115 (9.49)	599 (16.11)	0.19	115 (9.50)	131 (10.83)	0.044
I48	Atrial fibrillation and flutter	100 (8.26)	530 (14.251	0.19	100 (8.26)	111 (9.17)	0.032
J40-J47	Chronic lower respiratory diseases	94 (7.76)	407 (10.94)	0.11	94 (7.77)	102 (8.43)	0.024
E65-E68	Overweight, obesity and other hyperalimentation	77 (6.36)	463 (12.45)	0.21	77 (6.36)	70 (5.79)	0.024
I50	Heart failure	70 (5.78)	458 (12.32)	0.23	70 (5.79)	80 (6.61)	0.034
R53	Malaise and fatigue	69 (5.69)	292 (7.85)	0.086	69 (5.70)	68 (5.62)	0.0036
Z87.891	Personal history of nicotine dependence	58 (4.79)	322 (8.66)	0.15	58 (4.79)	66 (5.46)	0.029
F10.1	Alcohol abuse	57 (4.71)	156 (4.19)	0.025	56 (4.62)	55 (4.54)	0.0039
R63	Symptoms and signs concerning food and fluid intake	56 (4.62)	185 (4.97)	0.016	56 (4.63)	52 (4.29)	0.016
F10.2	Alcohol dependence	32 (2.64)	125 (3.36)	0.042	32 (2.65)	42 (3.47)	0.048
I73	Other peripheral vascular diseases	19 (1.57)	100 (2.69)	0.078	19 (1.57)	14 (1.157)	0.036
K74	Fibrosis and cirrhosis of liver	10 (0.83)	28 (0.75)	0.0082	10 (0.83)	10 (0.83)	0
1191	Aspirin	99 (8.18)	374 (10.06)	0.065	99 (8.18)	101 (8.35)	0.0060
11289	Warfarin	<10 (0.83)	66 (1.78)	0.084	<10 (0.83)	11 (0.91)	0.0089
1364430	Apixaban	<10 (0.83)	22 (0.59)	0.028	<10 (0.83)	<10 (0.83)	0
1114195	Rivaroxaban	0 (0)	<10 (0.27)	0.073	0 (0)	0 (0)	0

Table 2 shows outcomes after propensity score matching. 30-day survival rate was 0.9287 in cohort 1 vs 0.9536 in cohort 2, with a risk difference of 2.39% (95% confidence interval (CI) 0.557%-4.23%); relative risk (RR) 1.54, 95% CI (1.10-2.15); odds ratio (OR) 1.577, 95% CI (1.11-2.24); p=0.006. 90-day and 365-day end-point survival rates were not statistically different between cohorts. ICU level of care codes were billed an average of 9.76+-8.964 times in cohort 1 vs 14.618+-11.851 in cohort 2 (p<0.0001). At 365 days, there were no differences between the two groups for PE, MI, DVT, or palliative care consultation. Cohort 1 had fewer incidents at one-year (520) compared to cohort 2 (529). Cohort 1 had decreased incidence of pneumonia with 665 (54.95%) patients compared to cohort 2 with 725 (59.91%) (RR 0.917, 95% CI (0.856-0.983), OR 0.816, 95% CI (0.695-0.95), p = 0.013).

**Table 2:** Outcomes after propensity score matching PEG: percutaneous endoscopic gastrostomy

Outcome	Cohort 1, n (%)	Cohort 2, n (%)	Odds ratio (95% CI)	P-value
Mortality	219 (18.10)	221 (18.26)	0.989 (0.80-1.22)	0.92
PEG	520 (42.98)	529 (43.72)	0.97 (0.83-1.14)	0.71
Palliative care	138 (11.41)	159 (13.14)	0.851 (0.67-1.085)	0.19
Critical care days	9.76+-8.96	14.62 +-11.85	-	<0.0001
Pneumonia/pneumonitis	665 (54.95)	725 (59.92)	0.816 (0.695-0.959)	0.014
Pulmonary embolism	75 (6.20)	76 (6.28)	0.986 (0.71-1.37)	0.93
Myocardial infarction	55 (4.55)	72 (5.95)	0.753 (0.53-1.08)	0.12
Deep venous thrombosis	175 (14.46)	189 (15.62)	0.913 (0.73-1.14)	0.43

## Discussion

There is a debate over the best time to perform a tracheostomy on a ventilated patient with a severe stroke. In 2013, the Stroke-related Early Tracheostomy versus Prolonged Orotracheal Intubation in Neurocritical Care Trial (SETPOINT) trial examined patients with severe ischemic or hemorrhagic stroke who were expected to be on a ventilator for at least two weeks, and randomized patients into early versus late tracheostomy. They found that early tracheostomy did not decrease the average length of ICU stay, but did find decreased ICU mortality in patients with early tracheostomy, as well as decreased 6-month mortality [11]. This trial’s findings were in contradistinction to many previously reported findings of decreased ICU length of stay with early tracheostomy [6]. For example, the 2020 Collaborative European NeuroTrauma Effectiveness Research in Traumatic Brain Injury (CENTER-TBI) study looked at early (<=7 days) versus late (>7 days) tracheostomy in traumatic brain injury patients, and found that patients with late tracheostomy were more likely to have a worse neurological outcome, as well as a longer length of stay [12]. Likewise, a 2020 multicenter analysis looking at patients with myasthenic crisis found that early tracheostomy (performed before 10 days) was associated with shorter ventilation time as well as shorter duration of ICU stay for these patients as compared to late tracheostomy [13].

Similarly, a 2020 meta-analysis of early tracheostomy in severe traumatic brain injury patients showed that early tracheostomy reduced nosocomial adverse events, and allowed for early rehabilitation in these patients and early discharge, with associated reduced hospital and ICU length of stay. Patients who received earlier tracheostomies were charged approximately 306,422.85+-108,764.44 US dollars for their hospital course versus 345,925.98+-115,680.00 US dollars for the late tracheostomy group. The ICU cost was significantly reduced, at 13,623.47+-5,669.63 for the early tracheostomy group versus 21,261.25+-6,294.07 for the late tracheostomy group. Their mean ventilation days were 10.4+-0.28 versus 15.7+-0.57, mean ICU length of stay 14.6+-2.41 days versus 21.2+-2.50 days, and mean hospital stay 25.0+-1.18 days versus 33.02+-0.61 days [14].

Our results demonstrate that early tracheostomy (<= 7 days) versus late tracheostomy (>7 days) is associated with decreased length of ICU stay, decreased pneumonia/pneumonitis risk, but no improvement in survival outcomes at 90 and 365 days. There was no significant difference between groups for PE, MI, or DVT.

Our analysis was not without limitations. The major limitation of this study was that it was retrospective in nature. Furthermore, due to the nature of the database, we were unable to collect patient-level data on specific outcomes. The use of early vs. late tracheostomy timeline of 7 days, though arbitrary, is based upon the definitions in the literature and was established prior to the study. A meta-analysis indicated that studies indicating the endpoint of 7 days between early and late designation had better outcomes than studies that made the designation 14 or 21 days [15]. The data collected was for billing purposes, not for clinical use, and thus much clinical information is missing. There is a risk of selection bias in our cohort given that more critical patients will receive more intensive interventions earlier in their hospital course. In addition, some misidentification is inevitable in database studies.

## Conclusions

sICH is associated with high mortality rates, and those who recover are often left with a disability. Many of these patients require mechanical ventilation and eventual tracheostomy. In this study, we found that early tracheostomy in patients with sICH is associated with decreased pneumonia risk, decreased length of ICU care, and no difference in mortality at 90 and 365 days. This suggests that pursuing early tracheostomy in patients with sICH who are requiring mechanical ventilation without successful weaning may be an effective strategy for decreasing the ill effects of ventilator dependence, reducing hospital costs, and reducing in-hospital complications.
